# Restoring colistin sensitivity in colistin-resistant *Salmonella* and *Escherichia coli*: combinatorial use of berberine and EDTA with colistin

**DOI:** 10.1128/msphere.00182-24

**Published:** 2024-05-13

**Authors:** Xiao-die Cui, Xiao-kang Liu, Xiao-yuan Ma, Shuai-hua Li, Jun-kai Zhang, Rong-jia Han, Kai-fang Yi, Jian-hua Liu, Yu-shan Pan, Dan-dan He, Gong-zheng Hu, Ya-jun Zhai

**Affiliations:** 1 College of Veterinary Medicine, Henan Agricultural University, Zhengzhou, China; Hackensack Meridian Health Center for Discovery and Innovation, Nutley, New Jersey, USA

**Keywords:** Colistin, Berberine, EDTA, *Salmonella*, *Escherichia coli*

## Abstract

The appearance and prevalence of multidrug-resistance (MDR) Gram-negative bacteria (GNB) have limited our antibiotic capacity to control bacterial infections. The clinical efficacy of colistin (COL), considered as the “last resort” for treating GNB infections, has been severely hindered by its increased use as well as the emergence and prevalence of mobile colistin resistance (MCR)-mediated acquired drug resistance. Identifying promising compounds to restore antibiotic activity is becoming an effective strategy to alleviate the crisis of increasing MDR. We first demonstrated that the combination of berberine (BBR) and EDTA substantially restored COL sensitivity against COL-resistant *Salmonella* and *Escherichia coli*. Molecular docking indicated that BBR can interact with MCR-1 and the efflux pump system AcrAB-TolC, and BBR combined with EDTA downregulated the expression level of *mcr-1* and *tolC*. Mechanically, BBR combined with EDTA could increase bacterial membrane damage, inhibit the function of multidrug efflux pump, and promote oxidative damage, thereby boosting the action of COL. In addition, transcriptome analysis found that the combination of BBR and EDTA can accelerate the tricarboxylic acid cycle, inhibit cationic antimicrobial peptide (CAMP) resistance, and attenuate *Salmonella* virulence. Notably, the combination of BBR and EDTA with COL significantly reduced the bacterial load in the liver and spleen of a mice model infected with *Salmonella*. Our findings revealed that BBR and EDTA can be used as adjuvants collectively with COL to synergistically reverse the COL resistance of bacteria.

**IMPORTANCE:**

Colistin is last-resort antibiotic used to treat serious clinical infections caused by MDR bacterial pathogens. The recent emergence of transferable plasmid-mediated COL resistance gene *mcr-1* has raised the specter of a rapid worldwide spread of COL resistance. Coupled with the fact of barren antibiotic development pipeline nowadays, a critical approach is to revitalize existing antibiotics using antibiotic adjuvants. Our research showed that berberine combined with EDTA effectively reversed COL resistance both *in vivo* and *in vitro* through multiple modes of action. The discovery of berberine in combination with EDTA as a new and safe COL adjuvant provides a therapeutic regimen for combating Gram-negative bacteria infections. Our findings provide a potential therapeutic option using existing antibiotics in combination with antibiotic adjuvants and address the prevalent infections caused by MDR Gram-negative pathogens worldwide.

## INTRODUCTION

Global public health is now facing a formidable and growing menace from the emergence of multidrug-resistance (MDR) bacteria (1). Colistin (COL) has been used as a “last resort” against most members of the Enterobacteriaceae family, including *Escherichia coli*, *Salmonella* spp., and *Klebsiella pneumoniae* (2). Of special concern is the emerging resistance to COL, which can be mediated by chromosome alterations, plasmid-mediated *mcr* genes and multidrug efflux systems (3, 4). Combinations of existing antibiotics with adjuvants are emerging as a promising therapeutic approach to treat MDR bacterial infections (5, 6), and scholars are committed to exploring antibacterial adjuvants that exert non-specific enhancement effects by targeting efflux pumps and cell membrane (7, 8). Berberine (BBR), a natural isoquinoline alkaloid, has been involved in numerous investigations for its inhibition of efflux pump and moderate antimicrobial activity, especially against Gram-negative bacteria (9–11). EDTA is known to function as an uptake facilitator of outer membrane (OM) permeability, thus facilitating hydrophobic substrate uptake and enhancing the antibacterial activity against MDR bacteria (9, 12).

Recently, several strategies have been described to partially restore the bactericidal action of COL (13–15). However, there were no studies exploring the synergistic activity of BBR and EDTA in combination with COL against COL-resistant Gram-negative bacteria (GNB). Therefore, the primary objective of this research was to fathom the synergistic activity and the action mechanisms of BBR and EDTA in combination with COL against COL-resistant (COL-R) *Salmonella* and *E. coli*, and to provide new and possible treatment strategies for the treatment COL-resistant bacterial infections in the future.

## RESULTS

### BBR and EDTA exhibited significant synergy with colistin against COL-R *Salmonella* and *E. coli*

**Fig 1:**
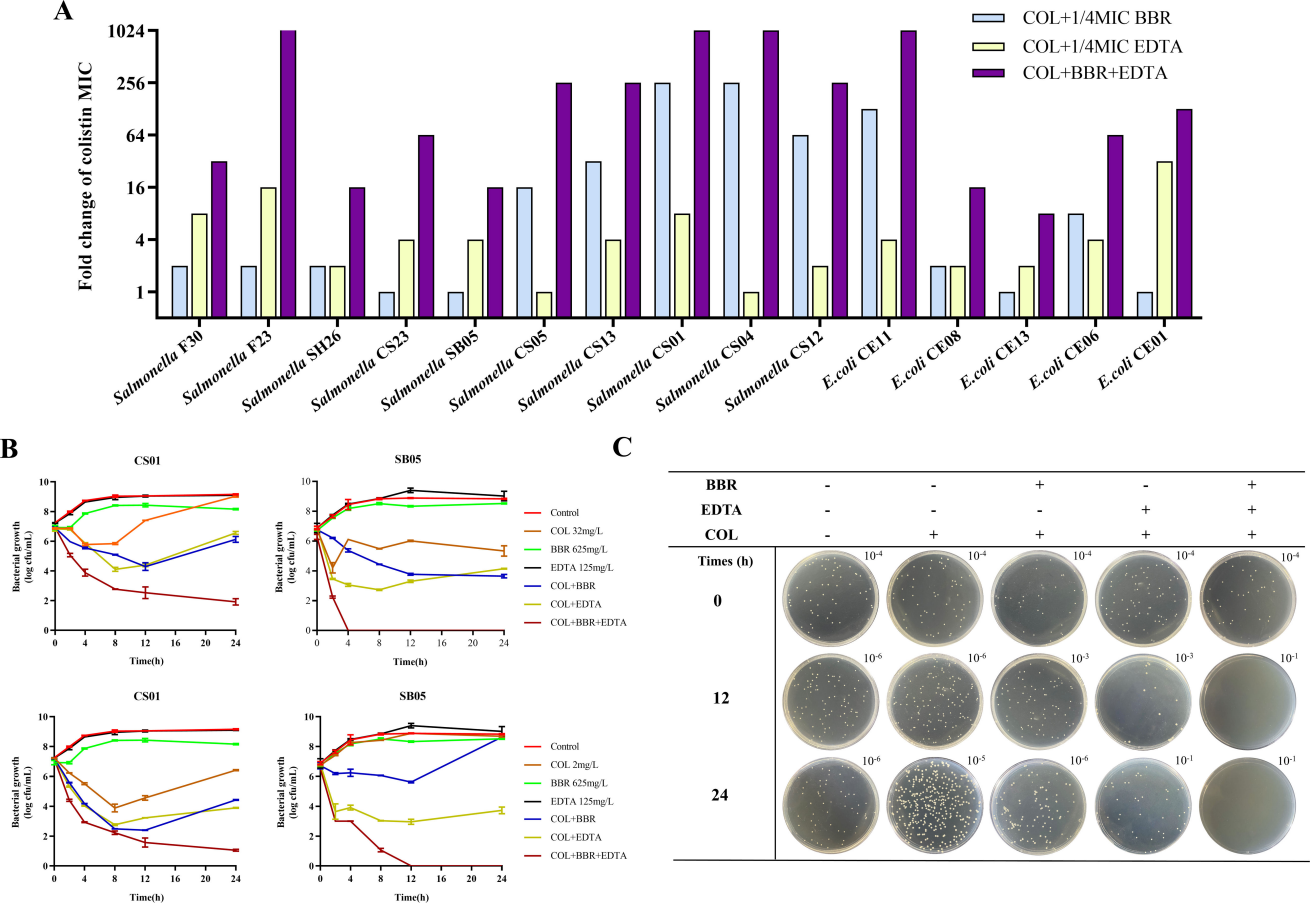
BBR combined with EDTA can significantly restore colistin sensitivity against colistin-resistant *Salmonella* and *E. coli*. (**A**) Fold change in MIC of COL in different strains after combination with BBR and EDTA. The bar graphs show the MIC values of COL for the tested bacterial strains when combined with 1/2 MIC of BBR and 1/2 MIC of EDTA or both. (**B**) Time-dependent killing curves of strains SB05 and CS01 treated with COL, BBR, and EDTA. One-half MICs of BBR for the two strains were both 625 mg/L; one-half MICs of EDTA for SB05 and CS01 were 125.0 and 62.5 mg/L, respectively. (**C**) Colonies on LB agar plates at the indicated time point in the time-dependent killing curve of *Salmonella* SB05 with COL of 2 mg/L.

As shown in Table 1, minimal inhibitory concentrations (MICs) of COL, BBR, or EDTA alone data indicated that BBR or EDTA showed no distinct inhibitory activity against all isolates, with the MICs of BBR of >625–1,250 mg/L and EDTA of >125–500 mg/L. Although 1/4 MIC BBR or 1/4 MIC BBR EDTA combined with COL (namely, BC and EC) could partly reverse the COL resistance of COL-R strains (MICs decreased by 1- to 256-fold), after 1/4 MIC BBR combined with 1/4 MIC EDTA (namely, BEC), the COL resistance phenotype was significantly reversed, with MIC of COL decreased by 8- to 2,048-fold (Fig. 1A). These results suggest that, despite the weak antibacterial effect of BBR or EDTA, BBR combined with EDTA exhibits synergistic potentiation with COL. Moreover, we speculated that BCE may function on broad-spectrum targets in these COL-R strains since their COL-R phenotypes were conferred by varied genetic alterations (Table S1). To further determine whether the synergistic potentiation was COL specific, we evaluated the synergistic effects of BBR and EDTA in combination with other antibiotics [olaquindox (OLA), mequindox (MEQ), ciprofloxacin (CIP), florfenicol (FFC), doxycycline (DOX), and gentamicin (GEN)] on the *mcr-1*-positive strain SB05. Generally, BBR combined with COL showed a stronger synergistic effect than the combination of EDTA and COL. One-half MIC BBR can reduce the MICs of various antibiotics, including OLA, MEQ, CIP, FFC, and DOX, while 1/2 MIC EDTA has no significant effect. However, compared with BBR alone, the combination of BBR and EDTA did not significantly reduce antibiotic resistance. Compared to other antibiotics, we found a better synergistic effect of 1/2 MIC BBR and 1/2 MIC EDTA combined with COL on SB05 strain, with MIC reduced by 128-, 128- and 32,768-fold reductions, respectively (Table 2).

**TABLE 1:** MICs and fold change of different combination strategies against COL-R *Salmonella* and *E. coli^a^*

Species	Name	COL (mg/L)	BBR (mg/L)					EDTA (mg/L)					BBR and EDTA (mg/L)			
			BBR	1/2 BC	FC	1/4 BC	FC	EDTA	1/2 EC	FC	1/4 EC	FC	1/2 BEC	FC	1/4 BEC	FC
*Salmonella*	F30	32	1,250	0.250	128	16	2	250	0.250	128	4	8	0.002	16,384	1	32
	F23	64	1,250	2	32	32	2	250	2	32	4	16	0.002	32,768	0.031	2,048
	CS23	32	1,250	0.500	64	16	2	125	8	4	16	2	0.031	1,024	2	16
	SB05	32	1,250	0.250	128	32	1	250	0.250	128	8	4	0.001	32,768	0.500	64
	SH26	4	1,250	0.500	8	4	1	125	0.250	16	1	4	0.031	128	0.250	16
	CS05	4	1,250	0.031	128	0.250	16	125	0.250	16	4	1	0.008	4,096	0.016	256
	CS13	16	2,500	0.063	256	0.500	32	250	0.016	1,024	4	4	0.0005	32,768	0.063	256
	CS01	32	1250	0.013	128	0.125	256	125	0.016	2,048	4	8	0.0002	131,072	0.031	1,024
	CS04	32	2,500	0.016	2,048	0.125	256	125	2	16	32	1	0.002	16,384	0.031	1,024
	CS12	16	1,250	0.031	512	0.250	64	250	0.063	256	8	2	0.0002	65,535	0.063	256
*E. coli*	CE11	16	1,250	0.016	1,024	0.125	128	125	0.500	32	4	4	0.001	16,384	0.016	1,024
	CE08	16	1,250	0.250	64	8	2	250	0.016	1024	8	2	0.002	8,192	1	16
	CE13	8	625	1	8	8	1	250	0.500	16	4	2	0.031	256	1	8
	CE06	4	625	0.250	16	0.500	8	125	0.250	16	1	4	0.016	256	0.063	64
	CE01	8	625	0.250	32	8	1	500	0.016	512	0.250	32	0.0005	16,384	0.063	128

^*a*^ Note: 1/2 BC, 1/2 EC, 1/4 BC, and 1/4 EC indicate 1/2 and 1/4 MICs of BBR (625.0-, 312.5-, or 156.25-mg/L BBR) or EDTA (250.0-, 125.0-, or 62.5-mg/L EDTA) combined with COL, respectively; 1/2 BEC and 1/4 BEC indicate 1/2 and 1/4 MICs of BBR or EDTA combined simultaneously with COL. COL, colistin; BBR, berberine; FC, fold change.

**TABLE 2:** MICs of several antibiotics combined with 1/2 MIC BBR or EDTA against *Salmonella* SB05^*a*^

Antibiotics	Alone	BBR (mg/L)		EDTA (mg/L)		BBR and EDTA (mg/L)	
		1/2 BC	FC	1/2 EC	FC	1/2 BEC	FC
OLA	256	16	16	128	2	8	32
MEQ	64	2	32	64	1	2	32
CIP	1	0.063	16	1	1	0.063	16
FFC	16	2	8	8	2	1	16
DOX	32	4	8	32	1	4	8
GEN	0.500	0.25	2	0.50	1	0.25	2

^*a*^ Note: 1/2 BC and 1/2 EC indicate 1/2 MIC of BBR or 1/2 MIC of EDTA combined with antibiotics, respectively; 1/2 BEC indicates 1/2 MIC BBR and 1/2 MIC EDTA combined simultaneously with antibiotics. BBR, berberine; CIP, ciprofloxacin; DOX, doxycycline; FC: fold change; FFC, florfenicol; GEN, gentamicin; MEQ: mequindox; OLA, olaquindox.

To further confirm the above synergistic effects of BBR, EDTA, and COL, time-kill assays with COL at 32 and 2 mg/L for SB05 (*mcr-1*-positive) and CS01 (*mcr-1*-negative) strains were performed (Fig. 1B). Overall, the combination of BBR, EDTA, and COL demonstrated stronger antibacterial effects than BBR and EDTA combined with COL separately. In the treatment of *mcr-1*-positive strain SB05 with the combination of the three drugs, all the bacterial colonies were killed within 24 h, effectively reducing the colony count by approximately four orders of magnitude compared to the treatments with BBR and EDTA combined with COL separately . Interestingly, we found a slight inhibition of BBR in the *mcr-1*-positive strain SB05 during 0–4 h incubation, but the inhibition was not observed for the *mcr-1*-negative strain CS01. Additionally, except for the similar growth trends of the two strains after the addition of BC and EC, we noticed that the BEC showed stronger antibacterial activity against *mcr-1*-positive strain SB05 than the *mcr-1*-negative strain CS01. We suspected that the combination of BBR and EDTA may inhibit the expression of *mcr-1*, which plays an assisting role in enhancing the bactericidal action of COL. The results of the checkerboard assay also demonstrated that compared to the combinations of BBR and EDTA combined with COL separately [fractional inhibitory concentration index (FICI) = 0.2539–0.75], the combination of COL + EDTA + BBR exhibited strong synergistic effects (FICI = 0.127–0.375) against all *Salmonella* and *E. coli* isolates (Table 3).

**TABLE 3:** FICI of two-drug and three-drug combinations against *Salmonella* and *E. coli* isolates^*a*^

Species	Name	FICI		
		COL + BBR	Col + EDTA	Col + EDTA + BBR
*Salmonella*	SB05	0.5	0.5	0.1875
	SH26	0.625	0.5	0.25
	CS05	0.3125	0.5625	0.141
	CS13	0.28125	0.5	0.1328
	CS01	0.2539	0.375	0.1289
*E. coli*	CE11	0.2578	0.5	0.127
	CE08	0.75	0.75	0.3125
	CE13	0.625	0.75	0.375

^*a*^ Note: BBR, berberine; COL, colistin.

### BBR can interact with the efflux pump AcrAB-TolC system and the MCR-1 protein, and when combined with EDTA, it can downregulate the expression of *mcr-1* and *tolC* in *Salmonella*

**Fig 2:**
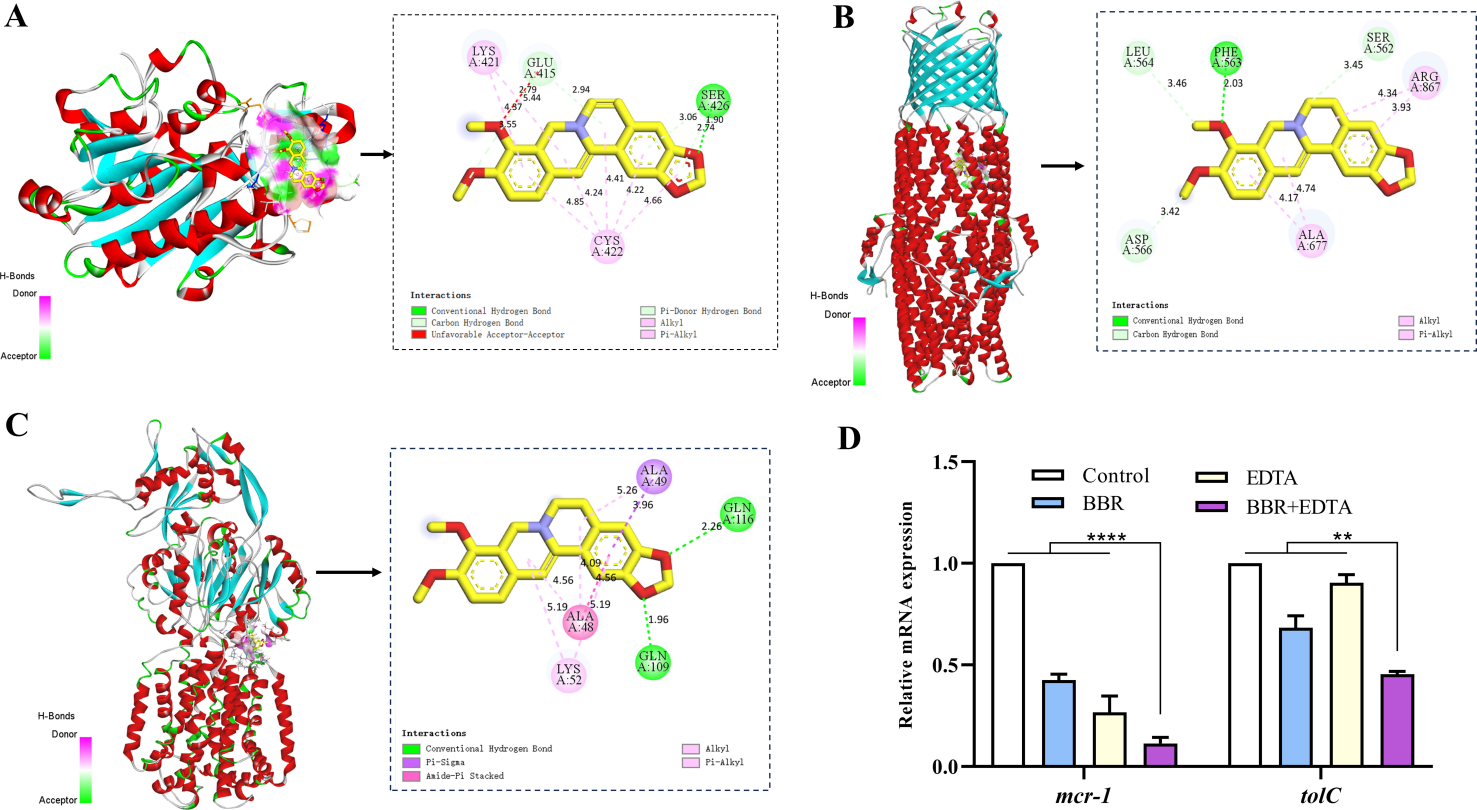
Prediction of binding modes of BBR with TolC, AcrB, and MCR proteins using molecular modeling. (**A–C**) Interaction determination of the BBR-MCR-1/TolC/AcrB complex by the molecular docking method. Docked pose of BBR is shown in the black box. The three-dimensional and planar binding interaction of a BBR molecule (ball and stick) with the surrounding amino acid residues (stick) is shown in the red dashed box. The conventional hydrogen bonding is represented by a dashed cyan line. (**D**) Expression of *mcr-1* and *tolC* in SB05 strain after treatment with COL BBR or both. The asterisks represent a significant difference (****P < 0.0001 and **P < 0.05) compared to the controlled expression level.

In order to further investigate the molecular mechanism, molecular docking simulations of the interaction between BBR and MCR-1, TolC and AcrB proteins were carried out, and the complexes of BBR/MCR-1, BBR/TolC and BBR/AcrB were obtained and shown in Fig. 2. We predicted possible interactions between three proteins and BBR, with the MCR-1 protein having a binding free energy of −8.04 kcal/mol with BBR. Among the simulated binding sites, a conventional hydrogen bond was formed with BBR at the Ser426 of the MCR-1 protein, which is regarded as an important residue for the binding interaction (Fig. 2A). The TolC protein of the efflux pump system had a binding free energy of −4.87 kcal/mol with BBR and formed one hydrogen bond, which is located at Phe563 of TolC protein respectively (Fig. 2B). The free energy of binding of AcrB protein with BBR was −4.87 kcal/mol, and two conventional hydrogen bonds were formed with BBR at the Gln116 and Gln109 position of AcrB protein (Fig. 2C). In view of previous reports that MCR-1 is a zinc metalloprotein, and EDTA as a strong metal chelator was related to a loss of MCR-1 activity and increased susceptibility to COL through stripping the metals from the protein (16). Furthermore, the expression of COL resistance gene *mcr-1* and OM channel protein *tolC* in strain SB05 were determined after 4 h of incubation with BBR, EDTA and COL alone or in combination. The results showed that when COL was combined with BBR or EDTA, the expression level of *mcr-1* and *tolC* were downregulated, but when BBR and EDTA were combined with COL, the expression of *mcr-1* and *tolC* were more significantly reduced. (Fig. 2D). Thus, our research suggested that BBR and EDTA could interact with MCR-1 protein, which further downregulate its transcription and activity, resulting in the increased susceptibility of *mcr-1*-positive strain SB05 to COL. In addition, BBR can likewise interact with AcrB and TolC proteins of the efflux pump AcrAB-TolC system to further regulate other efflux pump systems that use TolC as an OM channel protein.

### Colistin’s membrane-damaging ability is enhanced by BBR

**Fig 3:**
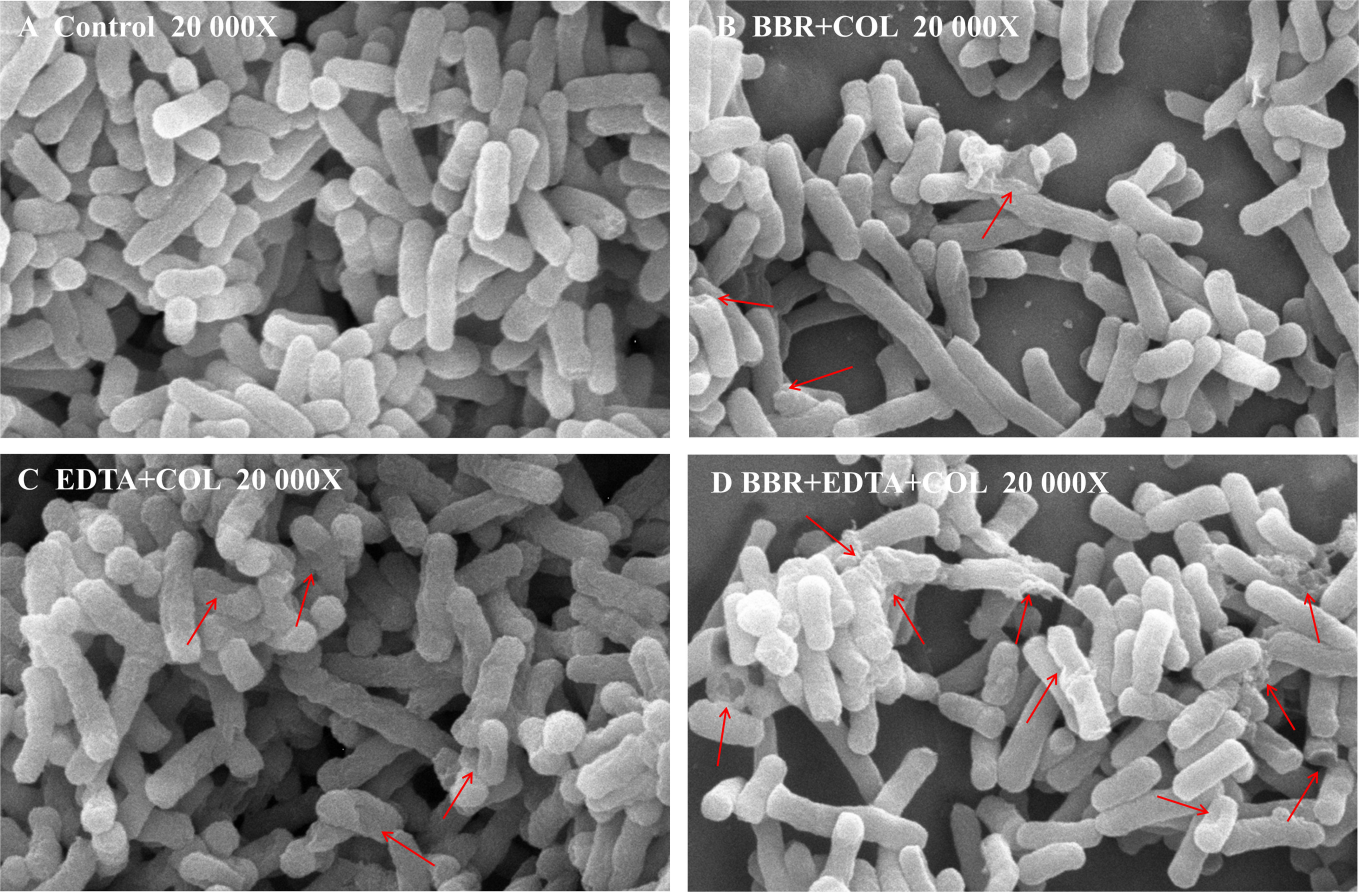
SEM images illustrating the morphological change of the bacterial cell membrane of *Salmonella* SB05 after various treatments. Arrows indicate bacteria with membrane damage or lysis.

Colistin has bactericidal activity against GNB by specifically interacting with lipopolysaccharides (LPS) in the bacterial OM, increasing the permeability of the cell membrane and causing cellular content leakage. Additionally, since EDTA is a known uptake facilitator of OM permeability (17), we hypothesize that the combination of BBR and EDTA can enhance the destructive effect of COL on cell membranes and rescue COL sensitivity. Next, scanning electron microscopy (SEM) analysis was applied to monitor the membrane damage induced by the combination of BBR, EDTA, and COL. The SEM results showed that, compared to the blank control group and the combination group of two drugs, more damage to the cell membrane and cell lysis were observed when COL was used in combination with BBR and EDTA (Fig. 3).

**Fig 4:**
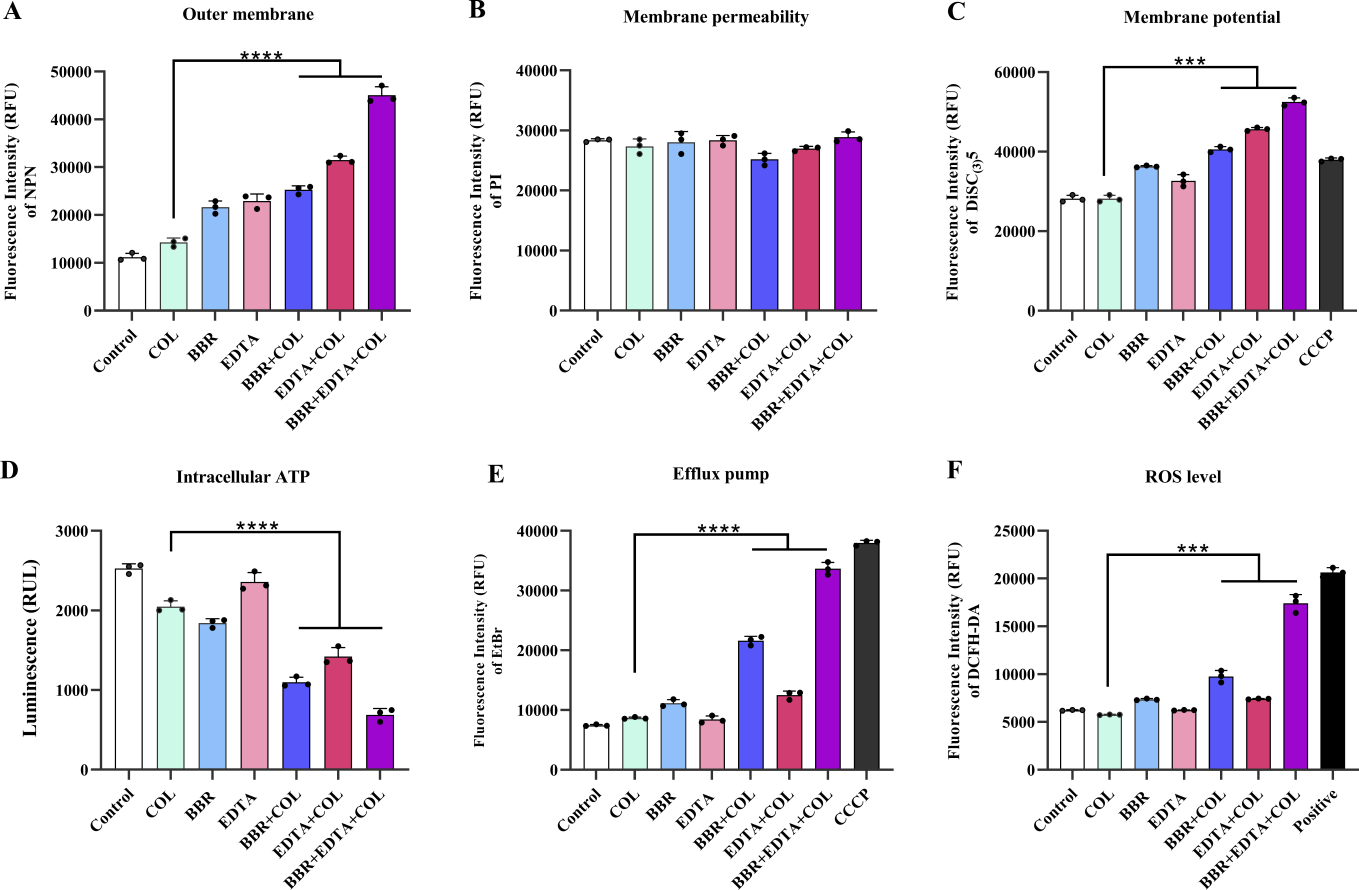
The mechanism of BBR combined with EDTA to restore colistin sensitivity. (**A**) The fluorescence intensity of NPN after 1.5-h exposure to BBR, EDTA, COL, and their combination was used to assess permeability. (**B**) No effect on membrane permeability for PI in *Salmonella* SB05 after treatment with BBR and EDTA. (**C**) BBR combined with EDTA significantly improves the effects of COL on the membrane potential of *Salmonella* SB05 while dissipating membrane potential. (**D**) *Salmonella* SB05 cells treated with BBR and EDTA produced less intracellular ATP surveyed by a luciferin-luciferase bioluminescence experiment. (**E**) Bacterial efflux pump activity in *Salmonella* SB05 after treatment with COL, BBR, EDTA alone, or their combination determined using ethidium bromide. (**F**) A fluorescence probe 2′,7′-dichlorodihydro-fluorescein diacetate (DCFH-DA) was used to monitor the levels of reactive oxygen species (ROS) in cells after exposure to COL, BBR, EDTA alone, or their combination. BBR combined with EDTA increased the level of ROS in *Salmonella* SB05.

In order to further investigate the ability of BBR combined with EDTA to enhance the damage of COL on bacterial cell membrane, we evaluated the effect of BBR combined with EDTA on the OM permeability and inner membrane integrity of *Salmonella* SB05 using the fluorescent probes N-phenyl-1-naphthylamine (NPN) and propidium iodide (PI). Compared with the effect of COL alone, both COL combined with BBR and COL combined EDTA can enhance OM permeability, while the combination of BBR and EDTA with COL led to significant enhancement of the permeability of the OM (Fig. 4A). In addition, the results of PI determination showed that BBR and EDTA had no significant effect on membrane permeability (Fig. 4B). These findings indicated that the combination of BBR and EDTA significantly enhanced OM permeability and membrane damage caused by COL in COL-resistant bacteria.

### The combination of BBR and EDTA inhibits efflux pump and promotes oxidative damage

After evidence that the combination of BBR and EDTA increases membrane disruption by COL, we then tried to investigate the other synergistic mechanism between COL, BBR, and EDTA. The effect of the addition of BBR and EDTA on the membrane potential (Δψ), which is a key component of proton motive force (PMF), was first examined using the 3,3-dipropylthiadicarbocyanine iodide [DiSC_3_(5)] probe. Our results indicated that the three-drug combination led to a significant increase in fluorescence values compared to single- and two-drug combinations, suggesting that the combination of BBR and EDTA disrupted the ΔΨ of *Salmonella* SB05, causing a dissipation of ΔΨ (Fig. 4C). Considering the high dependence of bacterial efflux pumps on PMF-driven ATP synthesis, we next evaluated the ATP levels in bacteria after exposure to BBR and EDTA, and investigated the effect of BBR combined with EDTA on efflux pumps using ethidium bromide (EtBr) dye. Consistently, a significant reduction in ATP levels was observed in *Salmonella* SB05 after incubation with BBR and EDTA (Fig. 4D), and the function of the bacterial efflux pump was also significantly inhibited, similar to the effect of the efflux pump inhibitor carbonyl cyanide 3-chlorophenylhydrazone (CCCP) (Fig. 4E). To further validate the role of inhibiting the efflux pump function in enhancing the efficiency of COL by BBR, we determined the synergistic activity of COL and BBR in the efflux pump gene *acrB* and *tolC*-deficient mutants of *Salmonella* standard strain CVCC541. The results demonstrated that the absence of *tolC* enhanced the synergistic activity of BBR and COL, indicating that the inhibition of efflux pumps is crucial for enhancing BBR’s potentiation of COL (Table S2). Membrane depolarization is also related to the production of reactive oxygen species (ROS), and the accumulation of ROS is usually accompanied by the corresponding decrease of intracellular ATP level (Fig. 4F). Therefore, further detection of ROS levels after BBR combined with EDTA was conducted to evaluate the oxidative damage status of *Salmonella* SB05. As expected, the combination of BBR and EDTA with COL significantly promoted the accumulation of intracellular ROS.

### BBR combined with EDTA accelerated the tricarboxylic acid cycle, inhibited CAMP resistance, and attenuated *Salmonella* virulence

**Fig 5:**
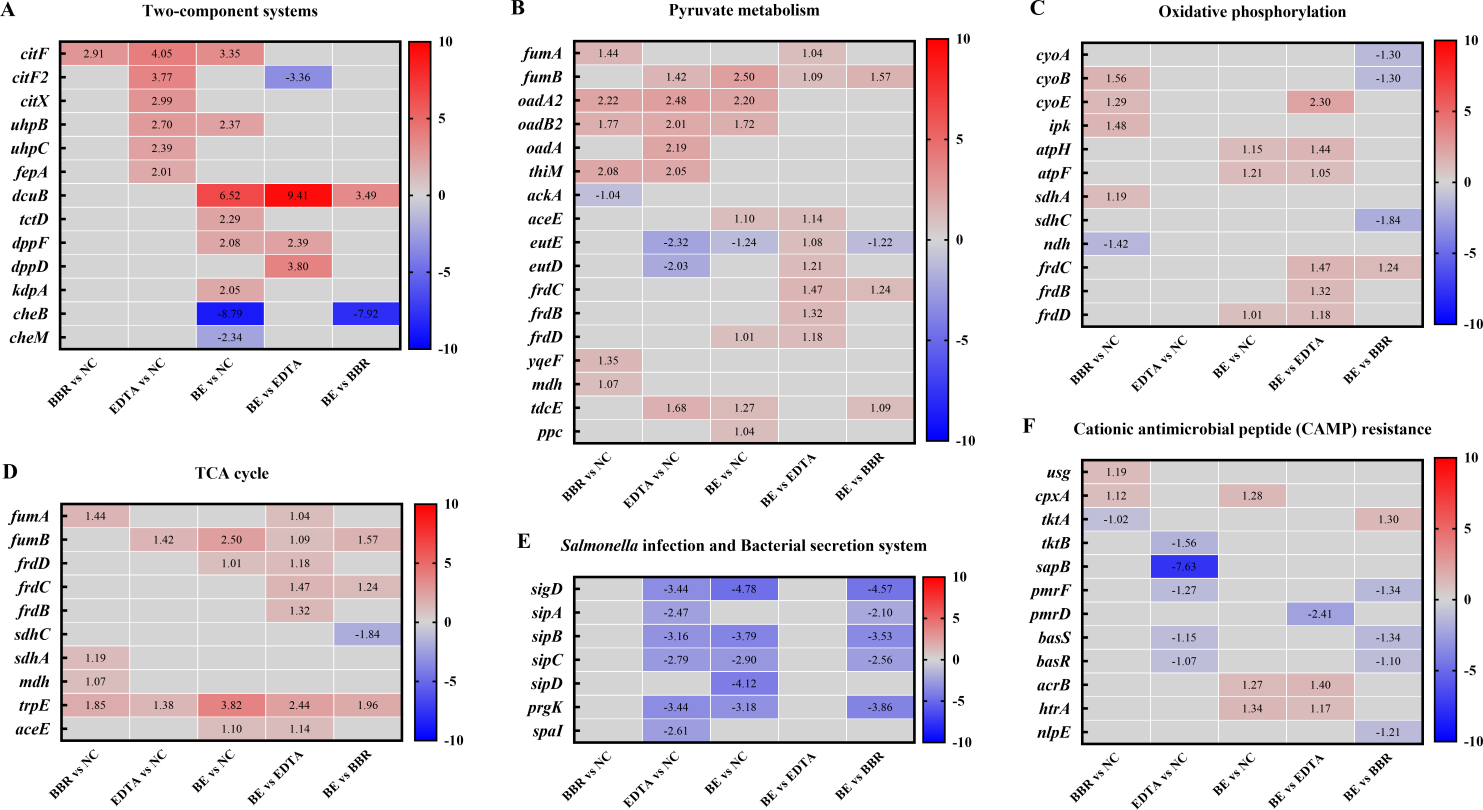
Transcriptional analysis of *Salmonella* SB05 treated by BBR and EDTA. Selected DEGs involved in *Salmonella* infection pathways, TCA cycle, pyruvate metabolism, oxidative phosphorylation, and CAMP resistance pathways. Red represents upregulation; blue indicates downregulation; and gray indicates the fold changes less than 1 or insignificant. BE, berberine combined with EDTA.

In order to investigate the more specific molecular mechanism of the BBR in combination with EDTA restoring COL sensitivity, transcriptional analysis was performed on *Salmonella* SB05 under treatment with BBR, EDTA or berberine combined with EDTA (BE) for 4 h. Differentially expressed genes (DEGs) were identified between five groups, BBR vs negative control (NC), EDTA vs NC, BE vs NC, BE vs EDTA, and BE vs BBR (Fig. 5). DEGs with a minimum twofold change (*P* < 0.05) in expression level are listed in the Table S2. Considering that BEC has better antibacterial effect than the combination of two drugs, we focused our attention on the DEGs of BE vs EDTA and BE vs BBR groups. Generally, these DEGs are mainly enriched in two-component systems (TCS), *Salmonella* infection pathways, tricarboxylic acid (TCA) cycle, pyruvate metabolism, oxidative phosphorylation, and CAMP resistance pathways (Fig. 5).

Specifically, we found that BBR combined with EDTA increased the expression levels of TCA cycle related genes (*fumA*, *fumB*, *frdC*, *frdB* and *frdD*). The TCA cycle is an integral part of the metabolism of bacteria. It has recently been shown as part of the newfound pyruvate cycle (the *P* cycle) (18). The TCA cycle and *P* cycle are energy-producing cycles in bacteria that promote the change of COL-resistant metabolomes to COL-sensitive metabolomes and eventually improve antibiotic efficacy (6, 18). In addition, the accelerated TCA cycle can enhance bacterial respiration and increase ROS production (19). Collectively, the combination of BBR and EDTA enhances oxidative damage in bacteria by accelerating the TCA cycle.

Of note, we accidentally found that the CAMP resistance related genes such as *pmrF*, *pmrD*, *basS*, *basR*, and *nlpE* were downregulated (1.10- to 2.41-fold) in the BE vs EDTA and BE vs BBR groups, as shown in Fig. 5F. The development of COL resistance mainly by reducing the negative charge of the OM, thus hinders the binding and the action of COL. Consistently, the downregulated DEGs in the CAMP resistance pathway may be closely related to the restored COL sensitivity, a cationic polypeptide antibiotic, after the combination of BBR, EDTA and COL. In addition, we found that the expression of the TCS gene *cheB* was significantly downregulated after BE incubation. CheB is known as a member of TCS CheA-CheW and is responsible for chemotaxis. Excepting for the *cheB* gene, other genes (*sigD*, *sipA*, *sipB*, *sipC*, and *prgK*) involved in the *Salmonella* infection and bacterial secretion system were also markedly downregulated after BE incubation (Fig. 5E), indicating that under BE stress, the SB05 strain changed their metabolism, struggling for survival, and meanwhile suffering fitness costs with reduction of adhesion, virulence, and invasion.

### BBR combined with EDTA enhances the bactericidal activity of colistin *in vivo*

**Fig 6:**
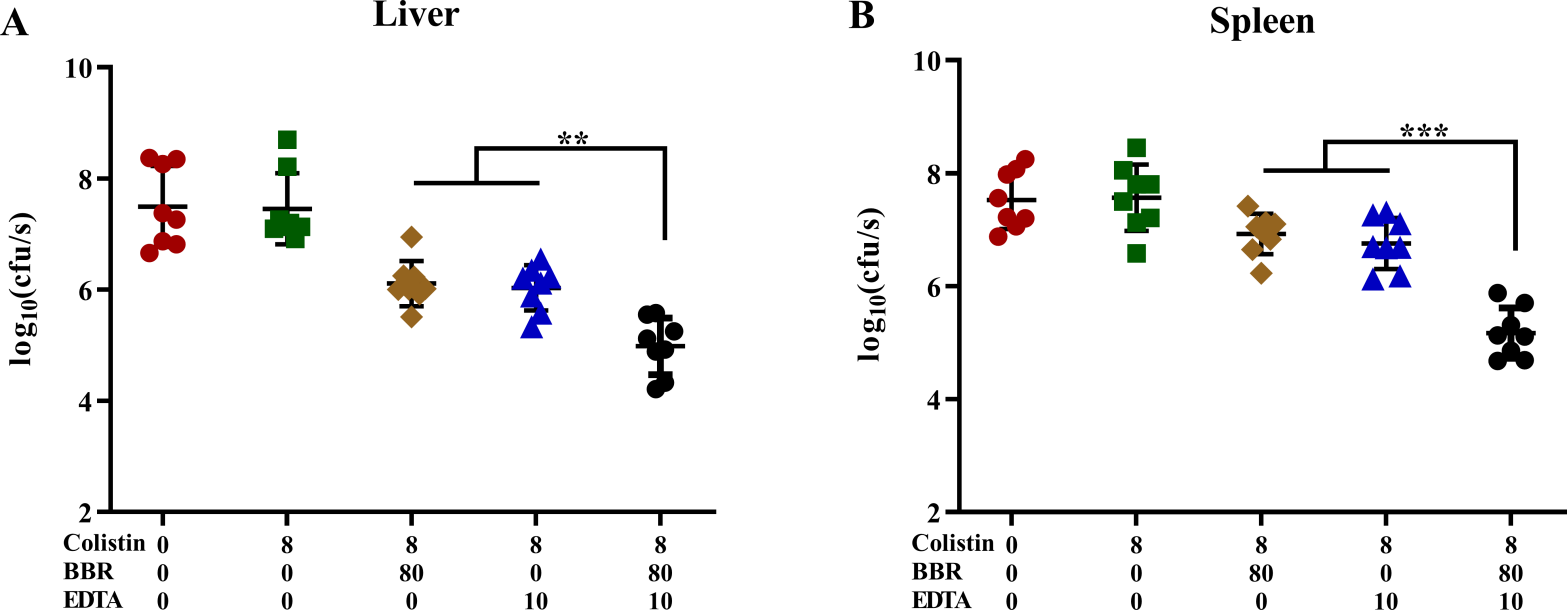
BBR and EDTA enhanced the antibacterial activity of colistin in a mouse infection model. The combined treatment of BBR reduced the *Salmonella* bacterial load in the liver and spleen of a mouse abdominal infection model. Balb/c mice (*n* = 8) were infected with a sub-lethal dose of 2 × 10^6^ CFU of *mcr-1*-positive *Salmonella* SB05 intraperitoneally and treated with separate COL (8 mg/kg), COL + BBR (8 + 80 mg/kg), COL + EDTA (8 + 10 mg/kg), COL + BBR + EDTA (8 + 80 + 10 mg/kg) and phosphate-buffered saline by intraperitoneal injection. *P* values, determined by Mann-Whitney *U* test (**P < 0.01 and ***P < 0.001), are reported. Data are presented as mean ± SD.

Encouraged by the significant synergistic bactericidal activity of BBR and EDTA combined with COL against GNB *in vitro*, we experimented the synergistic effect of BBR and EDTA on COL in a mouse infection model of *Salmonella*. The results showed that, compared to monotherapy, the combination therapy of COL with BBR or EDTA reduced the bacterial load in the liver and spleen of mice. Encouragingly, the three-drug treatment of COL, BBR, and EDTA showed a more significant reduction in bacterial load in the liver and spleen (Fig. 6). Specifically, the combinational therapy of COL, BBR, and EDTA (8 + 80 + 10 mg/kg) showed a decrease in bacterial load of approximately CFU≈2-log_10_ compared to monotherapy. This finding demonstrated that BBR restored COL sensitivity *in vivo*, and highlights the potential of BBR combined with EDTA as an antibiotic adjuvant to enhance bactericidal effect of COL against *Salmonella in vivo*. However, more promising clinical trials are still needed to validate the enhanced activity of the combination of baicalin and EDTA with COL *in vivo*.

## DISCUSSION

**Fig 7:**
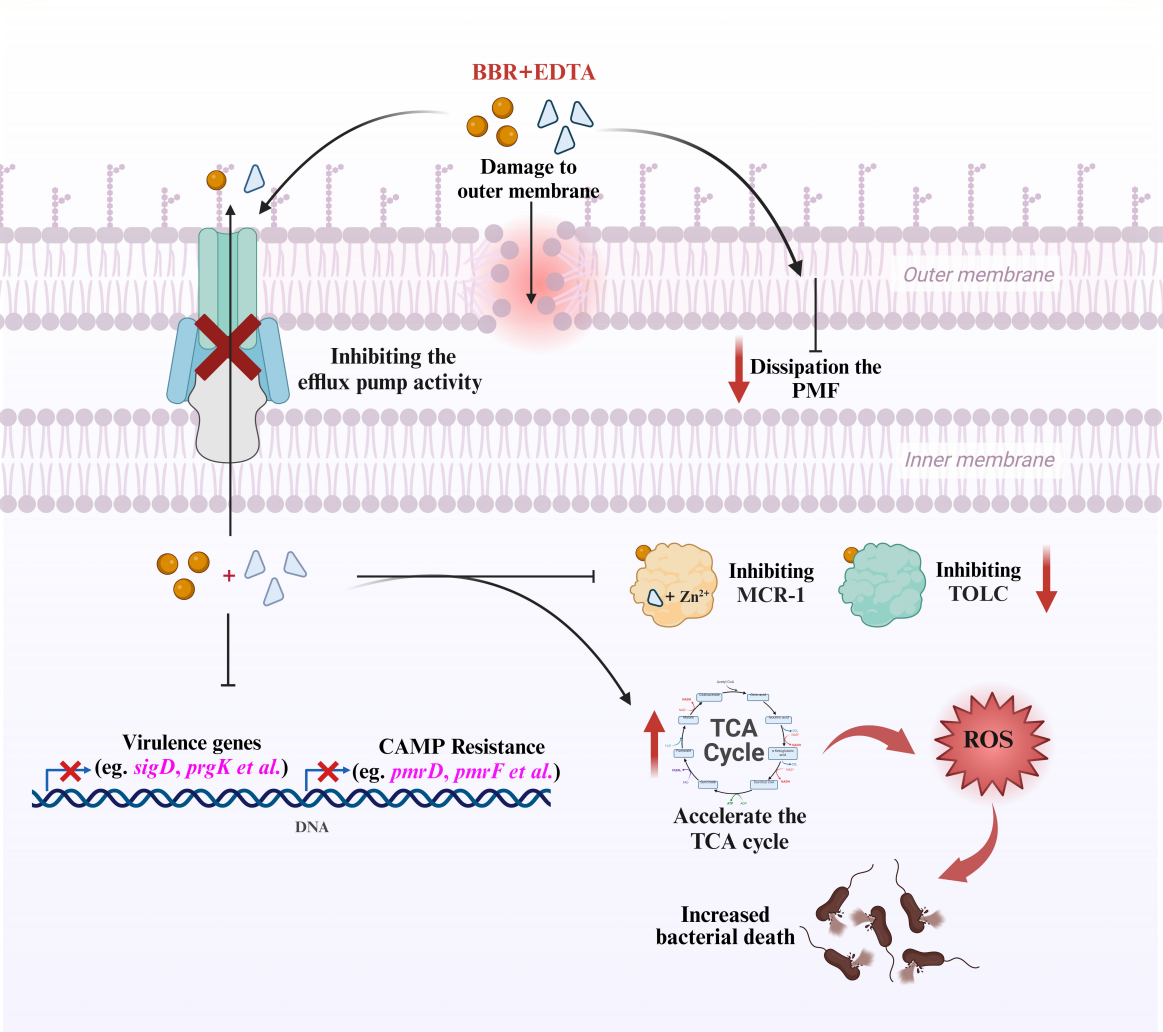
Schematic illustration of the mechanisms of BBR in combination with EDTA enhancing the antibacterial activity of colistin. The combination treatment of BBR and EDTA increases the permeability of bacterial OM, dissipates PMF in bacteria, inhibits the function of multidrug efflux pumps, enhances bacterial oxidative damage by accelerating the TCA, inhibits cationic CAMP resistance, and attenuates *Salmonella* virulence. In addition, BBR can directly interact with MCR-1 and TolC, and EDTA can chelate zinc ions in the active center of MCR-1, causing it to lose its activity. The combination of BBR and EDTA can significantly reduce the transcription of *mcr-1* and *tolC*.

Multidrug resistant (MDR) bacteria have emerged as a result of the widespread use of antibiotics to treat bacterial infections in recent years, and their rapid spread poses a severe threat to the world healthcare (20). Although being acknowledged as a last-resort antibiotic for the treatment of infections brought on by MDR, COL’s therapeutic effectiveness is significantly reduced by MCR-mediated COL resistance (21). In order to combat the infection of MDR bacterial pathogens, particularly Gram-negative pathogens, new treatment approaches are urgently needed (5). Natural compounds have attracted widespread attention as novel and safe antibiotics adjuvants. For example, proanthocyanidin isolated from cranberry has been demonstrated to increase the effectiveness of antibiotics, slow the development of resistance and help combat the increasing threat posed by antibiotic resistance (22). Nordihydroguaiac acid isolated from natural plants has also been proved to reverse the activity of COL and to strengthen the therapeutic effectiveness of COL (23). In this study, it was found that berberine can enhance the antibacterial activity of COL against *Salmonella* and *E. coli*, and this synergistic antibacterial activity is significantly enhanced through multiple molecular mechanisms when combined with the metal ion chelator EDTA (Fig. 7).

Berberine is a significant natural alkaloid, which has attracted mch attention due to its extensive and effective pharmacological activity. Synergistic antibacterial activity of berberine combined with other antibiotics such as sulbactam, meropenem and ciprofloxacin against MDR *Acinetobacter baumannii* has been reported (24). In addition, berberine can inhibit the efflux pump system of the major factor superfamily (MFS) and reverse the ciprofloxacin resistance in *E. coli* isolates (25). EDTA is a metal ion chelating agent that can increase the permeability of the OM and could lead to misfolded envelope proteins and activate the TCS CpxAR system. Previous studies have shown that the deletion of the efflux pump gene *acrB* and overexpression of *cpxR* (JS∆*acrB*∆*cpxR*/p*cpxR*) can significantly increase the susceptibility of *Salmonella* to COL (26, 27). Our research results also validated the experimental result mentioned above in clinically isolated *Salmonella*. To our knowledge, this research is the first report of synergistic activity of berberine and EDTA combined with COL in GNB. Encouragingly, BBR combined with EDTA was found to be effective in reversing COL resistance *in vivo* and *in vitro* via multiple modes of action, including increasing the damage of cell membrane, inhibiting efflux pumps, and increasing oxidative damage.

Given that the main interaction between COL and the bacterial surface is through the charge-based interaction with LPS, it is generally believed that the mechanism of COL resistance involves modification of LPS (28). However, recent reports suggest that the multidrug efflux pump plays a significant role in bacterial resistance to COL. In various bacterial species, the AcrAB-TolC, KpnEF, MtrC-MtrD-MtrE, VexAB, RosAB, and NorM efflux pumps have been considered to be related to COL resistance (29). The efflux inhibitor CCCP has been reported to reverse resistance to COL in a variety of bacteria, including *E. coli*, *K. pneumoniae*, and *Salmonella enterica* (30). This is consistent with our observation that berberine as a natural efflux pump inhibitor (25) can combine with EDTA to reduce the ATP levels in bacteria and inhibit the efflux pump function in *Salmonella*. Furthermore, the molecular docking analyses also predict that BBR can form hydrogen bonds with amino acid residues of AcrB and TolC of efflux pump system AcrAB-TolC.

Similar to the mechanism by which many natural compounds reverse COL resistance (22, 23, 31), BBR can also enhance cell membrane permeability and membrane damage of COL. EDTA is a typical antimicrobial and chelating agent, and it has always been used as a permeating and sensitizing agent which can combine with Mg^2+^ of LPS, resulting in LPS release and increased permeability of the cell membrane (32–34). The combination of BBR and EDTA has a stronger enhancing effect on cell membrane permeability and a stronger enhancing effect on the destruction of cell membranes by COL. The enhancement of COL activity by the combination of BBR and EDTA not only damages the cell membrane but also inhibits the multidrug efflux pump, induces the production of ROS and promotes oxidative damage . High levels of ROS induced by BBR and EDTA may lead to lipid peroxidation of cell membranes and further damage biological macromolecules such as cytoplasmic proteins and DNA, ultimately enhancing the killing effect of antibiotics on bacteria (35). In addition, our results indicated that the combination of BBR and EDTA can significantly reduce the Δψ of bacteria, leading to the dissipation of PMF. PMF itself is crucial for maintaining various key bacterial processes, such as ATP synthesis, flagella movement, and nutrient input (36), and impairment of this energy metabolism threatens the growth and survival of bacteria (37).

Although BBR as a single antibacterial agent has poor absorption and bioavailability (38), it still has accumulated a large amount of reliable therapeutic data and has developed new methods, such as nanoparticulate delivery system (39), to improve its bioavailability. In addition to new formulations, the development of new derivatives with similar biological activities based on the same mechanism but not limited by low pharmacological parameters seems to be the most important goal. Although our experimental results indicated that the combination of BBR with EDTA has a stronger synergistic effect against COL, the toxicity of EDTA prevents its clinical use. EDTA-derived compounds with lower toxicity but still retaining chelating activity may be used in combination with BBR to treat COL-resistant negative bacterial infections in clinical practice. According to reports, the low toxicity complex of EDTA and the calcium ion, calcium-EDTA (Ca-EDTA), significantly enhances the antibacterial activity of imipenem against *Pseudomonas aeruginosa* and can repair induced epithelial cell damage and acute lung injury in mice (40). This means that in the future, we may develop new delivery systems for BBR and Ca-EDTA to treat COL-resistant super bacterial infections.

### Conclusions

In conclusion, we demonstrated that BBR combined with EDTA is an effective colistin adjuvant that can reverse colistin resistance in *Salmonella* and *E. coli in vitro* and *in vivo*. Specifically, BBR combined with EDTA potentiates colistin activity via a variety of pathways, including increased cell membrane damage, destruction of PMF, inhibition of multidrug efflux pump function, and promotion of oxidative damage. Transcriptome analysis found that the combination of BBR and EDTA can accelerate the TCA cycle, inhibit CAMP resistance, and attenuate *Salmonella* virulence. In addition, BBR can interact with the MCR-1 protein and efflux pump system AcrAB-TolC, and the combination of BBR and EDTA can downregulate the *mcr-1* and *tolC* expression. Notably, colistin in combination with BBR and EDTA significantly reduced the bacterial load in the liver and spleen of mice model infected with *Salmonella*. Collectively, BBR combined with EDTA as a novel colistin adjuvant emphasizes the great potential of natural non-antibiotic compounds as potentiating antibiotics against bacterial infections.

## MATERIALS AND METHODS

### Bacterial strains

The 15 COL-R strains, including 10 *Salmonella* and 5 *E. coli* strains isolated from liver, spleen, and meat samples of chicken and swine, were included in this research. Whole-genome sequencing was performed for five strains, and we identified that the COL resistance phenotypes of these strains were mediated by the mutations of chromosomal-associated genes, such as *pmrAB*, *phoPQ*, *mgrB*, *pmrD*, *pmrC*, and *pmrHIJKLM*, as listed in Table S1. The efflux pump gene deletion strains of *Salmonella* CVCC541 are preserved strains in our laboratory.

### Antibiotic susceptibility testing

First, MICs of COL, BBR, or EDTA alone (simplified as Ca, Ba, or Ea) to the 15 COL-R strains were determined by broth microdilution method according to the Clinical and Laboratory Standards Institute (41) guidelines, and *E. coli* ATCC 25922 was used as the quality control (41). Then 1/2 or 1/4 MIC of BBR and EDTA were added to evaluate their combined bacteriostatic effect on these strains, namely, BBR + EDTA, BBR + COL, EDTA + COL, and BBR + EDTA + COL (simplified as BE, BC, EC, and BEC, respectively). Finally, MICs of olaquindox, mequindox, ciprofloxacin, florfenicol, doxycycline, and gentamicin (alone or in combination with BBR or EDTA) to *Salmonella* SB05 strain were determined.

### Time-kill assays

Time-kill assays of *Salmonella* SB05 (*mcr-1* positive) and *E. coli* CS01 (*mcr-1* negative) were performed in Mueller-Hinton broth with 1/2 MIC of BBR (625 mg/L), 1/2 MIC of EDTA (125 or 62.5 mg/L), COL (32 or 2 mg/L), BE, BC, EC, and BEC. Samples and a control group (drug-free) were simultaneously incubated with shaking at 37℃. The samples (100 µL) were taken out at defined time points (0, 2, 4, 8, 12, and 24 h) then serially diluted (10-fold) and plated on LB agar. After 16-h incubation, the log_10_ of viable cells (CFU/mL) was determined by time-kill curves. All experiments were tested in triplicate on different days.

### Checkerboard assays

Synergistic activity of COL and EDTA and BBR was evaluated by checkerboard assays. Each well is inoculated with 50 µL of 5 × 10^5^ CFU/mL test strain suspension, and the final volume is 100 µL. When the three drugs were combined, EDTA and BBR were diluted in a reaction well at the same time. The concentration ratio of EDTA and BBR was the optimal concentration ratio selected in the above-mentioned combined drug susceptibility test. The FICI was calculated according to the formula as follows: FICI = MICab/MICa + MICba/MICb = FICa + FICb. MICa is the MIC of compound A alone; MICab is the MIC of compound A in combination with compound B; MICb is the MIC of compound B alone; MICba is the MIC of compound B in combination with compound A; and FICa is the FIC of compound A, and FICb is the FIC of compound B. The FICI of ≤0.5 had a synergistic effect; 0.5 < FICI ≤ 1 had an additive action; FICI of >2 resulted in antagonism; and FICI of 1–2 had no interaction. The data were obtained in at least three independent experiments (42).

### Molecular docking

Crystal structure of MCR-1, TolC, and AcrB was obtained from the Protein Data Bank with PDB IDs 5GRR, 6WXI, and 2W1B, respectively. The three-dimensional structure of BBR was downloaded from the Pubchem Project (PubChem CID:2353). Molecular docking of the interaction between MCR-1, TolC, and AcrB with BBR was performed via AutoDock software. After the simulation, the affinity of the ligand/MCR-1 complex was measured to assess docking: the stronger the binding, the lower the parameter. Finally, the binding interactions and the binding sites were analyzed by applying Discovery Studio molecular graphics system.

### Quantitative real-time PCR

The transcriptional level of the *mcr-1* gene in *Salmonella* strain SB05 was analyzed in the presence of 1/4 MIC BBR, EDTA or BE. Total RNA was extracted following the phenol-chloroform method from cultures at OD_600_ ≈ 0.6, and the 16S rRNA gene was chosen as a housekeeping gene (26). The expression levels of *mcr-1* in samples with BBR, EDTA, or BE were calibrated with that without drugs.

### Transcriptome analysis

Transcriptome analysis was performed for the *Salmonella* SB05 strain treated with 1/4 MIC BBR, EDTA, or BE. *Salmonella* SB05 was grown overnight in LB broth and diluted 1:100 into 50-mL fresh LB supplemented with 1/4 MIC BBR, EDTA, or BE, and then incubated with shaking at 37°C for 4 h. Cells were washed three times with PBS and collected by centrifuging at 5,000 r.p.m for 5 min. Finally, thalluses were frozen in liquid nitrogen and sent to Beijing Tsingke Biotechnology Co., Ltd. for transcriptome analysis.

### Scanning electron microscope

Briefly, S*almonella* SB05 cells were cultured grown to logarithmic growth phase in LB broth, with final concentration of either 1/2 MIC COL, 1/4 MIC BBR, 1/4 MIC EDTA, or their combination in LB broth and incubated under rocking at 37°C for 6 h. At the end of the incubation, the bacterial cell particles were washed twice with PBS and then fixed with 2.5% glutaraldehyde at 4°C for 2 h. Fixed cells were then sequentially dehydrated with 30%, 50%, 70%, 90%, and 100% ethanol and air dried at room temperature. Scanning electron microscopy (Tescan FEI) was used to observe cell morphology.

### Biochemical factor measurement

The bacterial pretreatment in all biochemical measurements was carried out using a similar scheme as follows. *Salmonella* strain SB05 was grown overnight and diluted 1/100 in fresh LB followed by incubation with agitation at 37°C for 4 h. Then, 1/2 MIC COL (16 mg/L), 1/4 MIC BBR (312.5 mg/L), 1/4 MIC EDTA (62.5 mg/L), BC, EC, and BEC were added to the bacterial culture, respectively, and the shock culture was continued for 4 h. The *Salmonella* culture was washed and suspended in 5-mM HEPES (pH 7.0, plus 5-mM glucose) with an OD_600_ of 0.5, and different fluorescence dyes were added. The cells were incubated for 1.5 h, after which fluorescence units were measured on a Spapk 10 M Microplate reader (Tecan).

#### Permeability of outer membranes

The integrity of the OM of *Salmonella* SB05 was evaluated using the fluorescent probe NPN. Fluorescence units were determined with the excitation wavelength set to 350 nm, and the emission wavelength was set at 420 nm.

#### Cellular membrane integrity

After being treated with increasing doses of various medicines, 10-nM PI-labeled bacterial cells’ fluorescence intensity was evaluated using a 535-nm excitation wavelength and a 615-nm emission wavelength.

#### Membrane depolarization assay

The fluorescent probe DiSC3(5) (0.5 M) was used to assess the membrane potential of *Salmonella* SB05 (Aladdin, Shanghai, China). Using CCCP (1 mg/L) as a positive control, we measured *Salmonella* SB05 dissipated membrane potential using an excitation wavelength of 622 nm and an emission wavelength of 670 nm.

#### Efflux pump assay

The fluorescence dye EtBr (5 M) was used to assess the inhibitory effect of BBR and COL on the activities of efflux pumps. As a positive control, the well-known efflux pump inhibitor CCCP (1 mg/L) was used. The efflux of EtBr from bacterial cells was measured using an excitation wavelength of 530 nm and an emission wavelength of 600 nm.

#### Total ROS measurement

A 2′,7′-dichlorodihydro-fluorescein diacetate (10 µM) fluorescence probe (Beyotime, Shanghai, China) was used to measure ROS levels in *Salmonella* SB05 treated with BBR, COL, or the combination of these two compounds. Fluorescence intensity was tested after incubation with an excitation wavelength of 488 nm and an emission wavelength of 525 nm.

### ATP determinations

Intracellular ATP levels of *Salmonella* SB05 were measured with the use of an Enhanced ATP Assay Kit (Beyotime) assay. The BBR-, EDTA-, and COL-treated bacterial culture was centrifuged at 12,000 × *g* and 4°C g for 5 minutes, and the supernatant was removed. Bacterial precipitates were lysed with lysozyme and centrifuged, and the supernatant was ready for the determination of intracellular ATP levels. A 96-well plate with the detecting solution added was incubated for 5 min at room temperature. Following that, the supernatants were quickly added to the well and mixed. The Infinite M200 Microplate Reader was used to monitor the luminescence of supernatants (Tecan). The luminescence signals were used to calculate intracellular ATP levels in *Salmonella* SB05.

### Mouse intraperitoneal infection model

Murine infection assays were modified according to the reference described methods (38). Briefly, groups of Balb/c mice (*n* = 8) were intraperitoneally infected with 2 × 10^6^ CFU of *mcr-1*-positive *Salmonella* SB05 at a sub-lethal dose. Mice in the control group were injected with COL (8 mg/kg), BBR (80 mg/kg), EDTA (10 mg/kg), COL + BBR (8 + 80 mg/kg), COL + EDTA (8 + 10 mg/kg), or a combination of the three drugs (8 + 80 + 10 mg/kg). All of the mice were sacrificed 48 h after infection, and their weighted livers and spleens were deposited in 1 mL of sterile PBS on ice. After being homogenized, serially diluted, and plated on *Salmonella* Shigella agar, they were then cultivated at 37°C for 18–20 h to determine the number of bacteria loading.

### Statistical analyses

Statistical analysis was performed using SPSS version 26.0 and GraphPad Prism version 7.0 software. All data were presented as mean ± SD. Statistical assessments were performed using unpaired two-tailed *t*-tests, one-way analysis of variance (ANOVA), two-way ANOVA, or log-rank tests. Differences at a *P* value of <0.05 were considered significant. Significance levels are indicated with asterisks: *P < 0.05, **P < 0.01, ***P < 0.001.

## SUPPLEMENTAL MATERIAL

Supplemental Tables msphere.00182-24-s0001.docx Tables S1 and S2; caption for Table S3.

Table S3 msphere.00182-24-s0002.xlsx Genes with log2 fold change > 2 in transcriptome sequencing.

ASM does not own the copyrights to Supplemental Material that may be linked to, or accessed through, an article. The authors have granted ASM a non-exclusive, world-wide license to publish the Supplemental Material files. Please contact the corresponding author directly for reuse.
