# Supplementary material for: Restoring colistin sensitivity in colistin-resistant Salmonella and Escherichia coli: combinatorial use of berberine and EDTA with colistin
Source: mSphere. 2024 May 13;9(6):e00182-24. doi: 10.1128/msphere.00182-24 (PMC11332338; doi:10.1128/msphere.00182-24)
Supplement: Supplemental Tables — Tables S1 and S2; caption for Table S3. [file msphere.00182-24-s0001.docx]

**Table S1**. Sequencing results of colistin resistant genes in the whole genome sequencing of strains used in this study.

| Species | Isolate | Source/Reference | Mechanism of resistance to COL |
| --- | --- | --- | --- |
| *Salmonella* | F30 | Swine | *mcr-1* |
|  | F23 | Swine | *mcr-1* |
|  | SH26 | Swine | *mcr-1* |
|  | CS23 | Chicken | *mcr-1, eptB* (T84K, A657V) |
|  | SB05 | Swine | *mcr-1*, *mgrB* (Y31D), *eptB* (T84K, A657V) |
|  | CS05^a^ | Chicken | *pmrI* (Q48L, S195A, I655V), *pmrK* (M160T, L353I, V354I, K404Q, I455V), *pmrM* (F43L, Q65H), *pmrC* (L77P, A159V, G232S, E415Q), *pmrH* (N102T) |
|  | CS13 | Chicken | unknow |
|  | CS01^a^ | Chicken | *pmrI* (Q48L, S195A, M247I)*, pmrF*(T31A)*, pmrJ* (K54R, S164P, A268T)*, pmrK* (M160T, L353I, V354I, K404Q, I455V)*, pmrM* (F43L, Q65H), *pmrC* (L77P, A159V, G232S, E415Q), *pmrH* (N102T) |
|  | CS04^a^ | Chicken |  |
|  | CS12 | Chicken | unknow |
|  | CVCC541 | - | - |
|  | CVCC5412Δ*acrB* | (1) | CVCC541 gene-deleted strain |
|  | CVCC541Δ*tolC* | (2) | CVCC541gene-deleted strain |
| *E.coli* | CE11 | Chicken | *mcr-1* |
|  | CE08 | Chicken | *mcr-1* |
|  | CE13 | Chicken | *mcr-1* |
|  | CE06 | Chicken | *mcr-1* |
|  | CE01 | Chicken | *mcr-1* |

Note: The letter “a” at upper right corner indicated the three isolates CS01, CS04 and CS05, which have been analyzed by whole genome sequencing. Amino acid changes compared to that of CVCC541 were described in the format (original amino acid, position, substituted amino acid). For example, a substitution at position 10 from alanine to cysteine should be represented as tyrosine to aspartic acid.

**Table S2.** The effect of efflux pump gene deletion on the synergistic activity of BBR and colistin.

| Strains | COL MIC (mg/L) | FICI |
| --- | --- | --- |
| CVCC541 | 0.8 | 0.375 |
| CVCC541Δ*acrB* | 0.8 | 0.3125 |
| CVCC541Δ*tolC* | 0.05 | 0.15625 |

REFERENCES

1. Zhai YJ, Huang H, Liu J, Sun HR, He D, Pan YS, Hu G. 2018. CpxR overexpression increases the susceptibility of *acrB* and *cpxR* double-deleted *Salmonella enterica serovar* Typhimurium to colistin. J Antimicrob Chemother. 73:3016-3024.

2. Zhang MK, Zhang MY, Liu SB, Yang YY, Zhai YJ, He DD, Wu H, Pan YS, Liu JH, Yuan L, Hu GZ. 2021. Double deletion of *cpxR* and *tolC* significantly increases the susceptibility of *Salmonella enterica serovar* Typhimurium to colistin. *J Antimicrob Chemother* 76:3168-3174.

**Table S3.** Genes with log2 fold change greater ＞ 2 in transcriptome sequencing.

Note：The Excel file named "Supplementary Table 3" is uploaded separately as supplementary material.
